# Quantitative magnetic resonance imaging of brain atrophy in a mouse model of Niemann-Pick type C disease

**DOI:** 10.1371/journal.pone.0178179

**Published:** 2017-05-24

**Authors:** John W. Totenhagen, Adam Bernstein, Eriko S. Yoshimaru, Robert P. Erickson, Theodore P. Trouard

**Affiliations:** 1 Biomedical Engineering Program, University of Arizona, Tucson, Arizona, United States of America; 2 Department of Pediatrics, University of Arizona, Tucson, Arizona, United States of America; 3 Department of Molecular and Cellular Biology, University of Arizona, Tucson, Arizona, United States of America; 4 BIO5 Institute, University of Arizona, Tucson, Arizona, United States of America; 5 Department of Medical Imaging, University of Arizona, Tucson, Arizona, United States of America; 6 McKight Brain Institute, University of Arizona, Tucson, Arizona, United States of America; International Centre for Genetic Engineering and Biotechnology, ITALY

## Abstract

In vivo magnetic resonance imaging (MRI) was used to investigate regional and global brain atrophy in the neurodegenerative Niemann Pick Type C1 (NPC1) disease mouse model. Imaging experiments were conducted with the most commonly studied mouse model of NPC1 disease at early and late disease states. High-resolution in vivo images were acquired at early and late stages of the disease and analyzed with atlas-based registration to obtain measurements of twenty brain region volumes. A two-way ANOVA analysis indicated eighteen of these regions were different due to genotype and thirteen showed a significant interaction with age and genotype. The ability to measure in vivo neurodegeneration evidenced by brain atrophy adds to the ability to monitor disease progression and treatment response in the mouse model.

## Introduction

Niemann Pick Type C (NPC) is a rare genetic neurodegenerative disease which currently lacks effective treatments, and is universally fatal with death occurring prior to adulthood in the majority of patients [1][2]. NPC disease is most commonly diagnosed in early childhood with symptoms including ataxia, dysarthria, dysphagia, vertical supranuclear gaze palsy, and progressive neurological decline. The primary cause of the disease is mutation of the *NPC1* gene, resulting in a lack of functional NPC1 protein. The precise functions of the NPC1 protein are a topic of recent studies[3][4][5], and are known to include cholesterol transport within cells throughout the body. The dysfunction of NPC1 protein in NPC disease causes impaired cholesterol trafficking leading to a buildup of cholesterol and glycolipids in cells. A small percentage of NPC cases are caused by defects in NPC2 protein, which has been found to work with NPC1 to transport cholesterol [6][7]. The current study focuses on NPC disease caused by *NPC1* gene mutations (NPC1 disease).

A number of MRI studies of NPC patients have reported abnormalities in white matter tracts throughout the brain, gray matter atrophy, enlargement of ventricles and reduced cerebellar volume [8][9–12][13][14][15]. A study of adult patients reported a pattern of cortical frontal lobe atrophy associated with psychiatric or cognitive symptoms, while patients with gait and movement disorders had more pronounced brainstem and cerebellar atrophy, and at late stages of the disease, diffuse atrophy was found throughout the brain [16]. Another study of adult NPC patients with MRI morphometry techniques described widespread alterations in white matter tracts but focal rather than widespread reductions in gray matter volumes [17]. Variability in clinical reports of MRI-visible brain atrophy is likely due to differences in disease severity associated with the over 240 separate mutations of *NPC1* which are currently known to cause disease [18]. Due to the low incidence of NPC disease, estimated at approximately 1 in 150,000 live births [19], large clinical treatment studies are prohibitively difficult, making the use of animal models a valuable component of NPC disease research.

A commonly studied mouse model of NPC1 disease, *Npc1*^*-/-*^ has a mutation in the *Npc1* gene which causes truncation of 11 of 13 transmembrane domains of the Npc1 protein and consequently, a complete lack of functional Npc1 protein [20]. The *Npc1*^*-/-*^ mouse model exhibits disease symptoms mimicking a severe infantile form of NPC disease, and the mice live to approximately 10 weeks of age [2] on the BALB/cNctr genetic background. The *Npc1*^*-/-*^ model has been used in many studies of NPC disease, including recent studies of promising therapies using miglustat and cyclodextrins [21–23]. The phenotype of the *Npc1*^*-/-*^ brain has been described with white matter abnormalities including hypomyelination and myelin degeneration [24] and atrophy including a progressive loss of Purkinje cell neurons in the cerebellum [25]. MRI studies of the *Npc1*^*-/-*^ mouse model in vivo have shown abnormal myelination and atrophy of brain structures including the cerebellum, but have not examined brain abnormalities across the entire brain [26–29].

In vivo MRI-based mouse brain atlases and templates have made it possible to determine volumes of multiple brain regions from 3-dimensional (3D) MRI without the need for manual image segmentation. Currently available preclinical MRI systems and 3D imaging techniques have allowed us to use MRI atlas-based volumetry techniques in the preclinical disease model of the *Npc1*^*-/-*^ mouse. The current study utilized high-resolution in vivo 3D MRI in combination with atlas-based segmentation to investigate whole brain and region specific brain atrophy in the *Npc1*^*-/-*^ mouse model of NPC1 disease.

## Materials & methods

All animal procedures were carried out under approval of the University of Arizona Institutional Animal Care and Use Committee (IACUC). *Npc1*^*-/-*^ and age matched wild-type control (WT) mice were studied at 3 and 9 weeks of age, corresponding to an early pre-symptomatic age and an end-stage near-death time point, respectively. The numbers of animals in each group are indicated in Table 1. Imaging was carried out on a 7T Bruker Biospec MRI system (Bruker Biospin Corp., Billerica MA), using a 72mm ID birdcage coil for excitation and a four element phased array surface coil for signal reception. Mice were anesthetized with 2% isoflurane gas and positioned with an animal holding system including bite bar and ear bars for head fixation within the imaging coils. Breathing rate was monitored throughout all experiments and body temperature was maintained at 37°C with a heated circulating water system and monitored with a rectal fiber optic probe. Images were collected with a 3D fast spin-echo sequence with the following parameters: TR = 1800 ms, ETL = 8, Echo Spacing = 10 ms, TE_eff_ = 40 ms, FOV = 30 x 17 x 9.6 mm^3^, 100 μm isotropic resolution, and scan time: 60:08 (min:sec). Mice were euthanized at the end of the study via CO_2_ exposure.

**Table 1:** Brain region volume*, effects and interactions in 3 and 9 week old Npc1^-/-^ and control mice.

Brain region	WT 3 wks (n = 5)**	*Npc1*^*-/-*^ 3 wks (n = 6)**	WT 9wks (n = 4)**	*Npc1*^*-/-*^ 9 wks (n = 5)	Age	Genotype	Interaction
					p-value**		
**Whole Brain**	458.3 ± 20.0	424.8 ± 12.3	477.2 ± 33.9	400.6 ± 12.0	1.000	<0.001*	0.317
**Hippocampus**	27.5 ± 0.9	27.4 ± 1.2	29.4 ± 1.2	24.7 ± 0.8	1.000	0.001*	0.004*
**Internal Capsule**	1.2 ± 0.1	1.1 ± 0.2	1.7 ± 0.2	1.0 ± 0.1	0.362	<0.001*	0.024*
**Corpus Callosum and External Capsule**	12.0 ± 0.6	14.2 ± 0.5	12.1 ± 1.2	12.2 ± 0.4	0.14	0.016*	0.049*
**Caudate and Putamen**	25.0 ± 1.5	23.1 ± 1.4	28.3 ± 1.1	19.2 ± 1.4	0.772	<0.001*	<0.001*
**Anterior Commisure**	0.8 ± 0.1	0.9 ± 0.1	0.8 ± 0.1	0.8 ± 0.1	1.000	0.089	1.000
**Globus Pallidus**	1.3 ± 0.1	1.5 ± 0.2	1.7 ± 0.2	1.2 ± 0.1	1.000	0.022*	<0.001*
**Thalamus**	24.3 ± 0.9	21.9 ± 1.0	25.8 ± 1.2	19.8 ± 0.5	1.000	<0.001*	0.013*
**Cerebellum**	58.4 ± 3.6	54.7 ± 2.0	63.1 ± 4.6	48.1 ± 2.4	1.000	<0.001*	0.020*
**Hypothalmus**	12.1 ± 0.6	12.0 ± 0.2	14.0 ± 1.2	12.5 ± 0.6	0.024*	0.072	0.343
**Inferior Colliculi**	5.3 ± 0.1	5.1 ± 0.2	5.5 ± 0.4	4.3 ± 0.4	0.532	<0.001*	0.023*
**Superior Colliculi**	8.9 ± 0.2	8.2 ± 0.3	9.0 ± 0.7	7.1 ± 0.2	0.133	<0.001*	0.043*
**Central Gray**	3.7 ± 0.3	3.3 ± 0.1	3.9 ± 0.4	3.3 ± 0.2	1.000	0.007*	1.000
**Neocortex**	155. 4 ± 6.4	134.5 ± 6.0	157.7 ± 6.9	127.5 ± 2.8	1.000	<0.001*	0.720
**Amygdala**	10.8 ± 0.4	10.5 ± 0.3	12.2 ± 1.1	9.9 ± 0.6	1.000	<0.001*	0.042*
**Olfactory Bulb**	27.1 ± 1.3	24.5 ± 1.0	30.0 ± 2.1	25.6 ± 1.5	0.139	0.001*	1.000
**Brain Stem**	51.7 ± 2.8	49.6 ± 1.6	56.7 ± 4.5	50.0 ± 2.8	0.738	0.025*	0.609
**Basal Forebrain Septum**	15.7 ± 0.9	14.4 ± 0.7	16.5 ± 1.3	14.9 ± 0.8	1.000	0.022*	1.000
**Fimbria**	0.9 ± 0.1	1.1 ± 0.1	1.2 ± 0.1	1.2 ± 0.1	0.017*	0.015*	0.648
**Rest of Midbrain**	12.2 ± 0.6	12.1 ± 0.5	13.0 ± 1.0	11.7 ± 0.5	1.000	0.045	0.422
**Ventricles**	4.0 ± 0.4	4.8 ± 0.3	4.9 ± 0.6	5.6 ±	0.122	0.0576	0.903

*Brain volumes are reported in mm^3^

**p-values listed are after Holm-Bonferroni correction for multiple comparisons

In the resulting images, brain tissue was semi-automatically segmented from non-brain tissue. An initial brain surface demarcation was made with an intensity based 3D region of interest (ROI) selection tool included in the MRIcron software package (http://people.cas.sc.edu/rorden/mricron/index.html). The brain edges were manually examined and adjusted in the coronal orientation for each slice of the 3D image datasets, with reference to a mouse brain atlas [30]. The brainstem was manually trimmed at the base of the cerebellum for each dataset. Heterogeneous signal intensity due to the use of a four element phased array surface coil was corrected by the use of the N4ITK bias correction method [31], as implemented in the Advanced Normalization Tools (ANTS) toolbox [32].

Following this, images were registered to an in vivo mouse brain MRI atlas [33], using the Symmetric Normalization (SyN) registration algorithm as implemented in the ANTS toolbox [32] using parameters appropriate for mouse brain registration [34]. Registration with the in vivo MRI brain atlas allowed for quantization of volumes from twenty brain regions to be calculated for each dataset without the need for manual drawings of ROIs.

Two-way ANOVA was performed on each brain region defined by the atlas in order to evaluate changes in volume due to both age and disease status. For all statistical analyses, a Holm-Bonferroni correction was used to correct for multiple comparisons with a significance level of 0.05.

## Results

**Fig 1:**
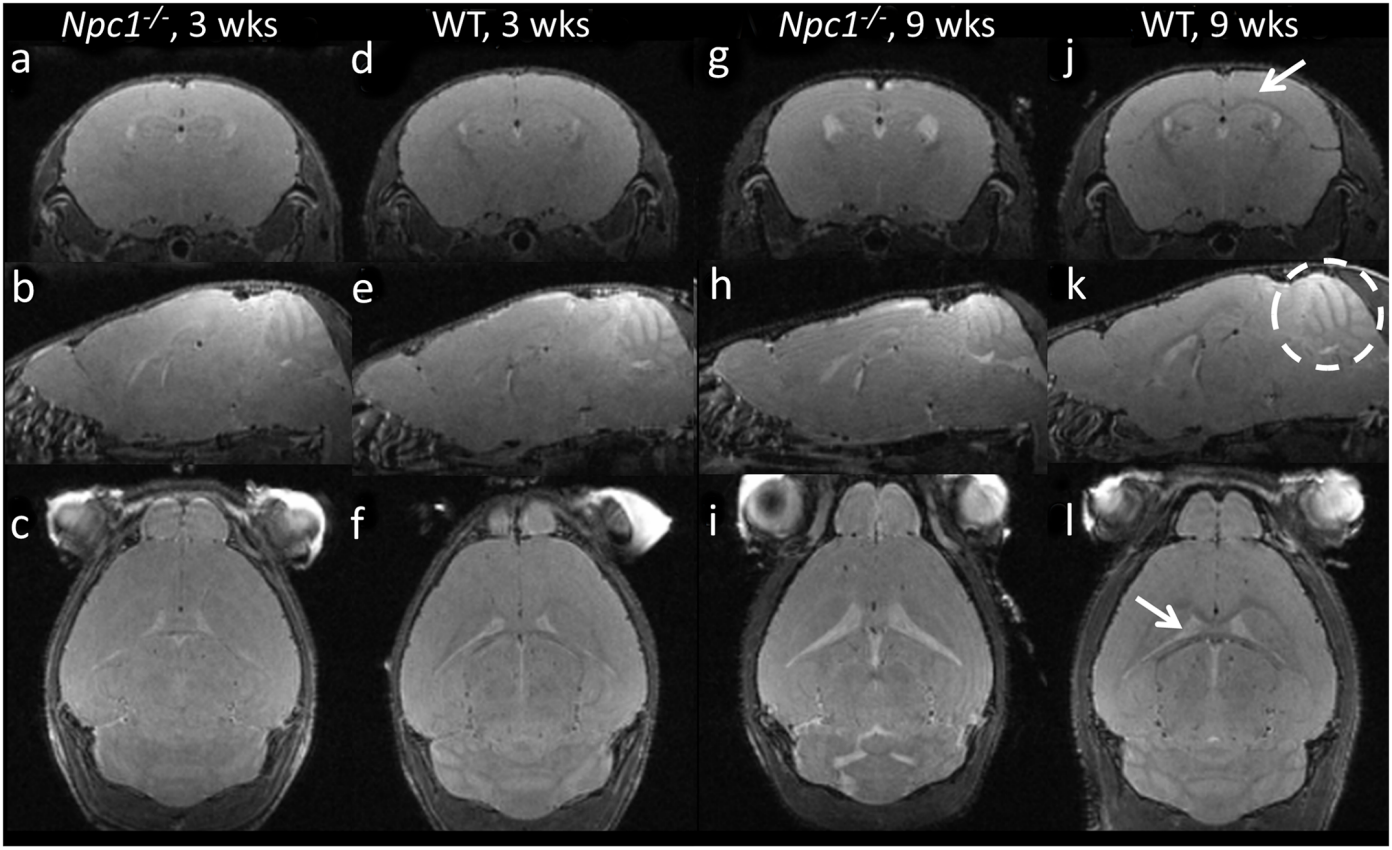
Example anatomical MRI images of mice at early and late timepoints. Example high resolution T2-weighted in vivo images of *Npc1*^-/-^ (a-c, g-i) and WT (d-f, j-l) mice at 3 weeks (a-f) and 9 weeks (g-l) of age. At three weeks of age there are only slight differences between in the brains of *Npc1*^-/-^ and WT mice, but can be seen in the white matter regions of the brain. These differences become more pronounced at 9 weeks of age. The arrow in panel j points to the region of the corpus callosum and external capsule in a WT mouse, which demonstrates a dark band of intensity compared to the surrounding gray matter. This is reversed in the *Npc1*^-/-^ mouse. The cerebellum is circled in panel *k*, which is visibly reduced in size in the *Npc1*^-/-^ mouse at 9 weeks. The arrow in panel *l* indicates the bright signal of the CSF in the lateral ventricles, which are increased in size in the *Npc1*^-/-^ mouse at 9 weeks.

Representative images of late stage (9 weeks) and early presymptomatic (3 weeks) *Npc1*^*-/-*^ mice and WT controls are shown in Fig 1. Differences in contrast between gray and white matter are visible in the white matter regions of the corpus callosum, external capsule, fimbria, and internal capsule. The cerebellum of the *Npc1*^*-/-*^ mouse appears reduced in size relative to the WT mouse, especially apparent in the sagittal section at 9 weeks of age. The overall size of the brain at 9 weeks of age also appears reduced in the *Npc1*^*-/-*^ mouse relative to WT.

**Fig 2:**
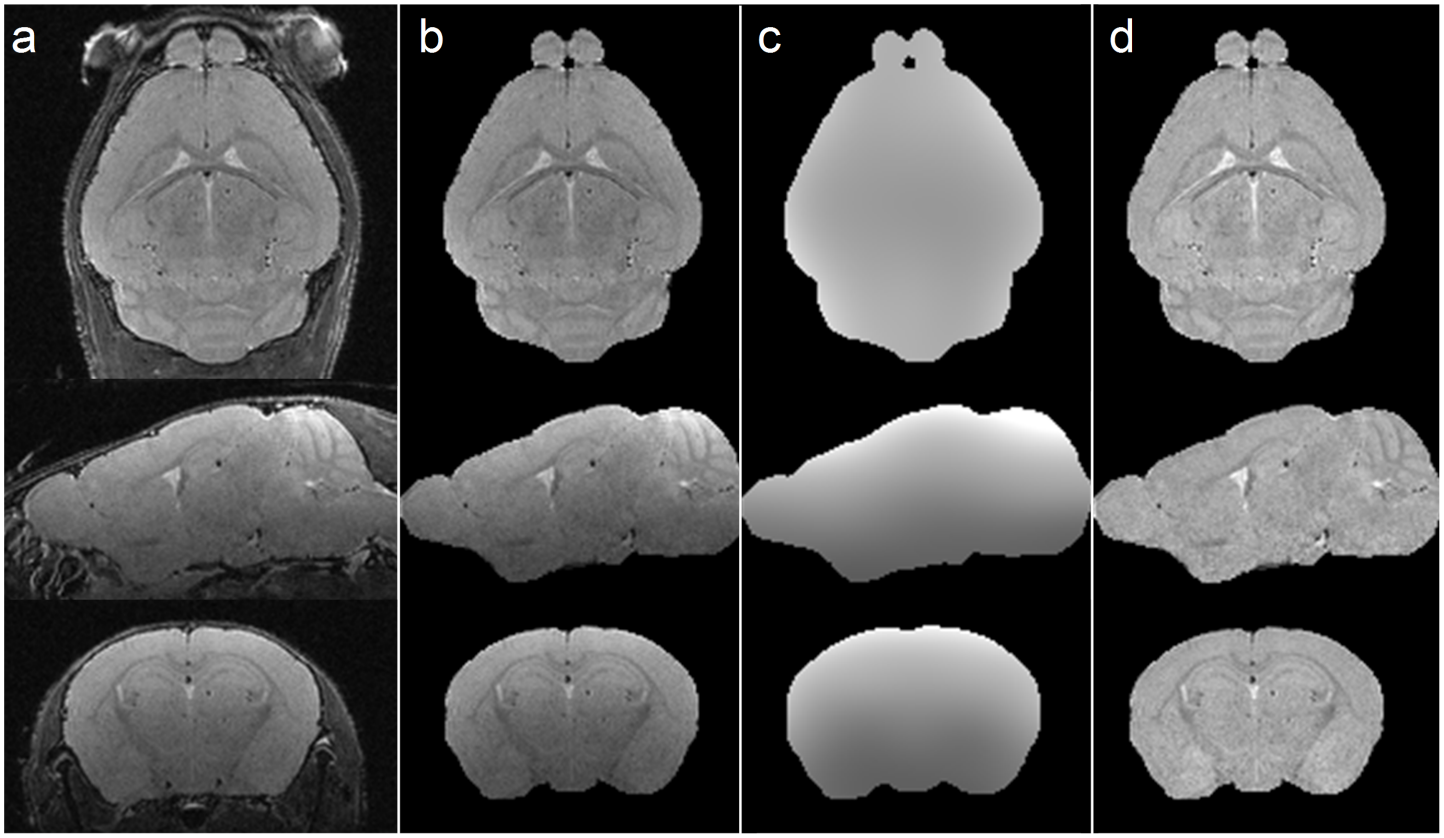
Example 9 week WT mouse images illustrating processing steps for volumetry analysis. Orthogonal views of the example T2-weighted in vivo dataset are shown in panel *a*. The images after semi-automated segmentation to remove signal from non-brain material are shown in panel *b*. The bias field estimate due to surface coil sensitivity inhomogeneity obtained from N4ITK software is shown in panel *c*. Panel *d* is the processed 3D dataset ready for image registration and analysis.

Fig 2 demonstrates the steps used in the processing of the high resolution T2-weighted datasets for analysis. Orthogonal sections from an individual WT mouse at 9 weeks of age are shown in Fig 2a. Fig 2b displays the result of the semi-automated masking of non-brain tissue from the 3D volume. The estimated bias field from the heterogeneous sensitivity of the 4-channel surface coil, as determined by the N4 technique, is shown in Fig 2c. The high signal intensities in the upper regions of the cortex and cerebellum due to surface coil sensitivity are visible as bright regions in the bias field. The segmented and bias field intensity-corrected images are shown in Fig 2d and are used for volumetric analyses.

**Fig 3:**
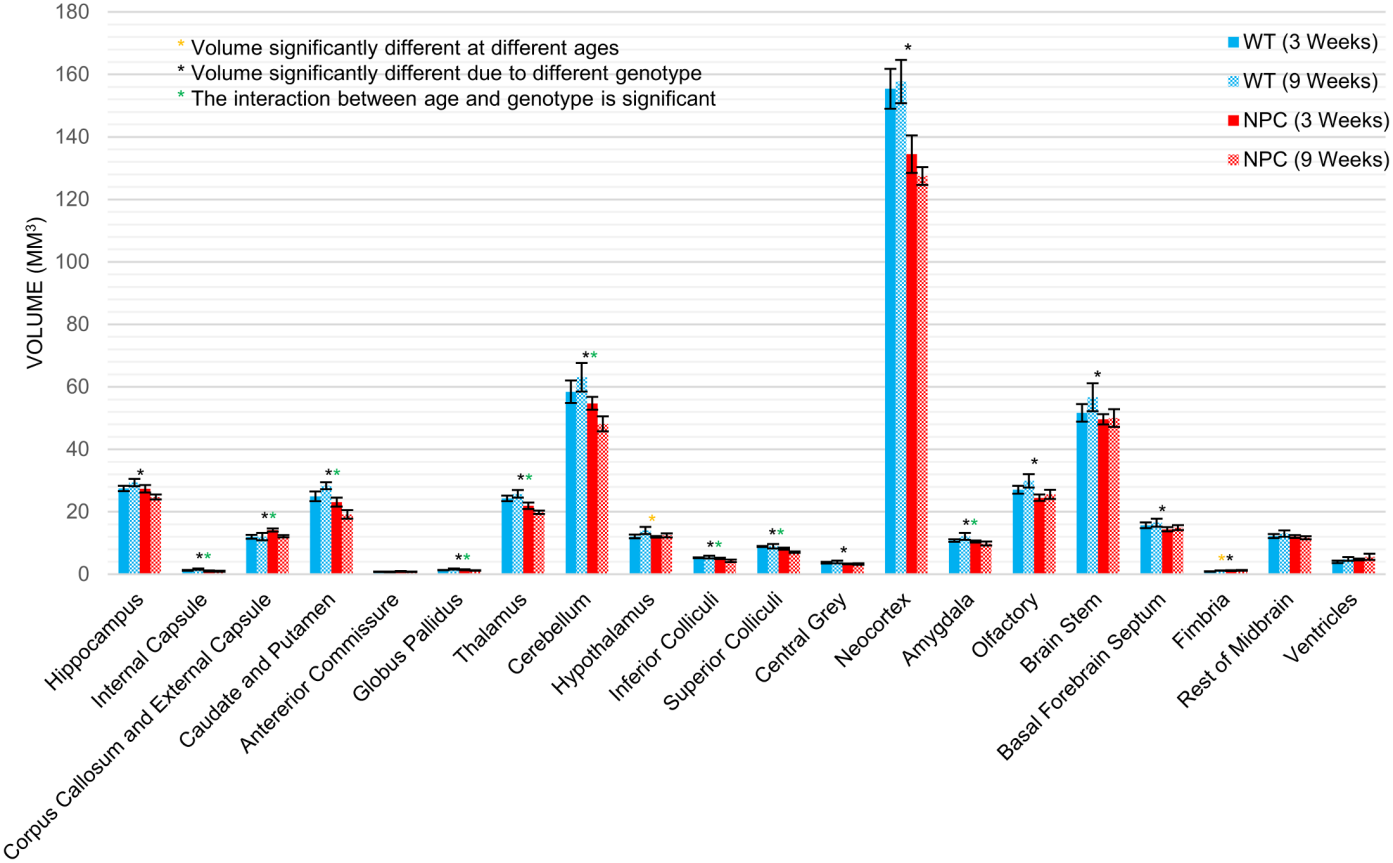
Results of whole-brain segmentation volumes. Volumes of twenty mouse brains regions are shown for WT mice (blue) and *Npc1*^-/-^ (red) mice at 3 (dark) and 9 (light) weeks of age. Error bars indicate the standard deviation within groups. Significant effects of genotype (*) and age (*) and significant interaction between age and genotype (*) were determined from a two-way ANOVA and p<0.05 after Holm-Bonferroni correction.

**Fig 4:**
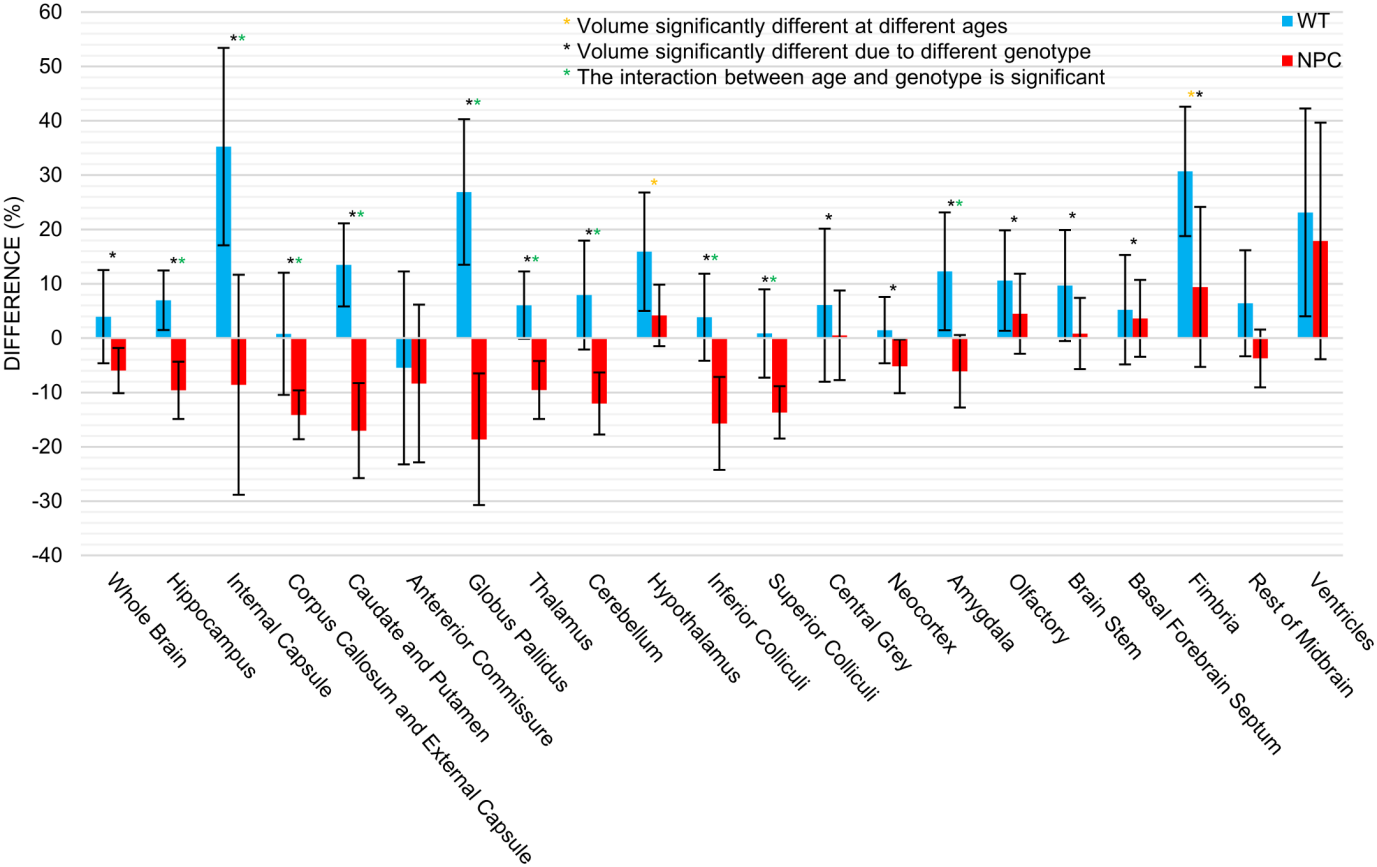
Change in the volume of mouse brain regions with age. Percent change in whole brain and brain region volumes between 3 and 9 months of age for WT (blue) and *Npc1*^-/-^ (red) mice. Errors bars indicate the standard deviation of the percent change. Significant effects of genotype (*) and age (*) and significant interaction between age and genotype (*) were determined from a two-way ANOVA and p<0.05 after Holm-Bonferroni correction.

Volumes of twenty brain regions and whole brain volume generated from atlas-based registration of WT and *Npc1*^*-/-*^ mice are listed in Table 1 and brain region volumes are plotted in Fig 3. Sixteen of the twenty brain regions identified showed a significant effect of disease status, i.e. genotype, on brain region volume. Two of the regions showed a significant effect of age on volume and ten of the regions showed a significant interaction between age and genotype. For most brain regions, volumes in *Npc1*^*-/-*^ mice at 3 weeks of age were smaller than the regions in WT mice. Notable exceptions to this are the ventricles and corpus callosum and external capsule. At 9 weeks of age, every brain region in *Npc1*^*-/-*^ mice was smaller than those in WT mice, except the ventricles, which remain larger in *Npc1*^*-/-*^ mice. In order to evaluate the change in brain region volume with age specifically, the percent change in brain region volume from 3 to 9 weeks of age is plotted in Fig 4. In WT mice, every brain region, except for the anterior commissure, increased in size from 3 to 9 weeks of age. The entire brain was increased from 458.3 to 477.2 mm^3^ and the cerebellum from 58.4 to 63.1 mm^3^. In *Npc1*^*-/-*^ mice, thirteen of the brain regions identified decreased in volume. The entire brain decreased from 424.8 to 400.6 mm^3^ and the cerebellum from 54.7 to 48.1 mm^3^. In the brain regions which increased with age in *Npc1*^*-/-*^ mice, the increase was always less than that of WT mice for the same region.

## Discussion

The high-resolution 3D MRI and semi-automated atlas-based registration carried out in the *Npc1*^*-/-*^ mouse model demonstrated differences in the volume of several brain regions in *Npc1*^*-/-*^ mice compared to WT mice at an early, pre-symptomatic stage of the disease and more pronounced differences at a late, end-stage of the disease. Significant effects of the disease were seen in several brain regions including the hippocampus, caudate and putamen, thalamus cerebellum, superior colliculi and neocortex. These brain regions have previously been reported to be affected by neurodegeneration in clinical NPC cases as well as in ex vivo studies of the *Npc1*^*-/-*^ mouse model and are the regions of highest levels of Npc1 mRNA [35].

Clinical case studies of NPC disease have described widespread but variable amounts of MRI visible brain atrophy associated with neurodegeneration. A study of adult NPC patients quantified brain atrophy with morphometric methods[17], and reported focal areas of grey matter reductions in the areas of the hippocampus, cerebellum, and thalamus. Similar regions were found to be significantly reduced in volume at 9 weeks of age in the current study of the *Npc1*^*-/-*^ mouse. Recent studies of NPC patients across a wide spectrum of ages and disease severities were able to correlated corpus callosal and cerebellar volume to disease severity[13][14]. Alterations in microstructural properties of white matter in these two regions were also measured. Neuroimaging case studies of adult NPC patients have also been carried out with ^18^F-flourodeoxyglucose positron emission tomography (FDG-PET), which provides a measure of tissue metabolic activity that can be associated with neurodegeneration. Reported FDG-PET findings include severe hypometabolism in the frontal cortex, thalamus, and cerebellum [36], which would be expected with neurodegenerative atrophy in those regions, similar to the current findings in the *Npc1*^*-/-*^ mouse. A second FDG-PET study found hypometabolism in the prefrontal cortex and thalamus, but hypermetabolism in the cerebellum hypothesized to be related to symptomatic dystonia [37].

An ex vivo MRI study reported reduced whole-brain and cerebellar volumes at 6 weeks of age [29]. In vivo MRI studies of the *Npc1*^*-/-*^ mouse to date have identified, but not quantified, atrophy in the brain [26–28,38]. We found a trend towards decreased cerebellar volume at 3 weeks which was highly significant at 9 weeks. Purkinje cell degeneration is detectable at 4 weeks [39] and is quite advanced by 6 weeks [40], but the volume of the cerebellum is more dependent on the number of granule cells [41] which do not undergo the last round of division [42]. Thus, the marked volume changes we found reflect failure of development of granule cells as well. This defective initial development of the cerebellum lead to delayed motor skill acquisition [43]and eventually results in motor in-coordination and tremors which have been abundantly described [44].

The decreased volume of the thalamus likely reflects the previously described neuronal loss in the thalamus, especially in the ventral posterior lateral and medial nuclei [45] while the relative lack of volumetric change in the brainstem may reflect the gliosis which is abundant there [46]. The neocortical volume loss we found correlates with the findings of a quantitative ex vivo study of regional brain volumes in 11 week old *Npc1*^*-/-*^ mice which used a stereological cell counting method and found significantly decreased numbers of neurons in the prefrontal cortex (as well as the thalamus)[47]. Memory loss [48] is a consequence of this neocortical and, also, the hippocampal volume losses.

The current work, while able to semi automatically measure in vivo brain volume differences in the *Npc1*^*-/-*^ model compared to WT, has limitations. With all atlas-based registration methodology the results will necessarily depend on how well the registration process worked and whether it worked the same on images from both experimental groups. Consistent image registration is assumed for analysis used in this work. While the ANTS program has been shown to be well suited to MRI brain registration tasks [32], and used often in mouse brain MRI evaluation [34][49][50], registrations of small structures with complex shapes such as white matter tracts and ventricular spaces could be subject to error. A major question is whether the difference in volume reported by the atlas-based registration is truly due to differences in volume, or to differences in the performance of the registration. While visual inspection indicates a qualitatively similar registration this does not guarantee that registration differences do not impact the volumes reported. This uncertainty, however, does not diminish the confidence in the statistical results that show brain regions in *Npc1*^*-/-*^ and WT mice are different. Because of the small error in the volumes reported in each group and at each age, we can be confident that differences between the brains are real, but these differences may be from more than a simple volume change, e.g. results could be impacted by changes in tissue signal from brain regions which might affect registration.

To our knowledge, this is the first study to utilize high-resolution volumetric MRI in conjunction with semi-automated atlas-based registration to evaluate changes in the brain of the *Npc1*^*-/-*^ mouse model at early and late disease stages of the disease. A significant effect of genotype on volume was observed in almost every brain structure analyzed. These regions correspond to findings reported in ex vivo studies of the *Npc1*^*-/-*^ model as well as clinical reports of NPC disease. The ability to measure in vivo neurodegeneration evidenced by brain atrophy provides an additional means to monitor disease status and could prove useful in future studies of treatments in the *Npc1*^*-/-*^ mouse model of NPC1 disease.

The *Npc1*^*-/-*^ mouse model studied in this work has been utilized extensively in NPC research but represents a severe, infantile form of the disease. It would be very interesting to compare the results of this study with similar studies of the *Npc1*^*nmf164*^ mouse model, which contains a point mutation similar to many human forms of the disease and has some NPC1 activity (37). The slower progression of the disease in the *Npc1*^*nmf164*^ mouse could result in greater "temporal separation" of the changes in differing brain regions which could allow delimitation of possibly differing pathological processes in them. Such changes could also serve as biomarkers for the success or failure of current and future therapeutic interventions.
